# A case of subdural hematoma with a medical history of hemophilia a and a review of related literature

**DOI:** 10.1186/s41016-018-0119-6

**Published:** 2018-05-23

**Authors:** Yi Zhao, Xin-Jie Bao, Yong Yao, Yuan-Fan Yang, Jun-Ji Wei, Wen-Bin Ma, Ren-Zhi Wang

**Affiliations:** Department of Neurosurgery, Peking Union Medical College Hospital, Chinese Academy of Medical Sciences, 1 Shuaifuyuan, Wangfujing, Beijing, 100730 China

**Keywords:** Intracerebral hemorrhage, Hemophilia, Factor VIII

## Abstract

**Background:**

Intracranial hemorrhage is the most common emergency in the neurology department, and patients with a medical history of hemophilia have a risk of severe bleeding.

**Case presentation:**

A 56-year-old man was admitted to the emergency department in our hospital. He was diagnosed with hemophilia A and subdural hematoma. We administered an infusion of factor VIII to decrease the risk of bleeding and improve the prognosis. Factor VIII infusion is the most important factor in treating hemophilia A patients.

**Conclusion:**

We recommend carefully checking coagulation function and the medical history once these patients are admitted, especially in the emergency department.

## Background

Intracranial hemorrhage (ICH) is a common emergency in neurology departments and includes many complications. Hemophilia is a rare complication that increases the risk of bleeding in conditions such as subdural hematoma (SDH), ICH, and subarachnoid hemorrhage (SAH). Acute SDH is a lethal disease with a high mortality rate, and the mortality of patients with hemophilia is also high [1]. Thus, improved monitoring and early treatment for SDH are more necessary than previously thought. Here, we report a case of SDH in a male patient with a medical history of hemophilia. We administered an infusion of factor VIII and provided adjunctive treatment during the perioperative period, successfully saving his life. In addition, we summarize the characteristics of treatment for SDH patients with a history of hemophilia A through a literature review.

## Case presentation

A 56-year-old man went into a coma after presenting with a headache and vomiting spontaneously 8 h before he was admitted to the hospital. The patient’s medical history was normal, except for hemophilia A. Due to a lack of factor VIII in the admitting hospital, he was transferred to our hospital.

On arrival, the patient’s Glasgow Coma Score was 6/15. The diameters of the left and right pupil were 4 mm and 6 mm, respectively. The light reflex was absent in the right eye and diminished in the left. The patient had hypermyotonia with an absence of myodynamia, and the Babinski sign was positive on both sides. Coagulation function was assessed. The activated partial thromboplastin time (APTT) was increased to 103.4 s, factor VIII inhibitor was 0, and fibrinogen was decreased to 1.64 g/L. A computed tomography (CT) scan showed right acute parietal and frontotemporal SDH with a 15-mm midline shift. The hematoma volume was approximately 60 ml.

**Fig. 1:**
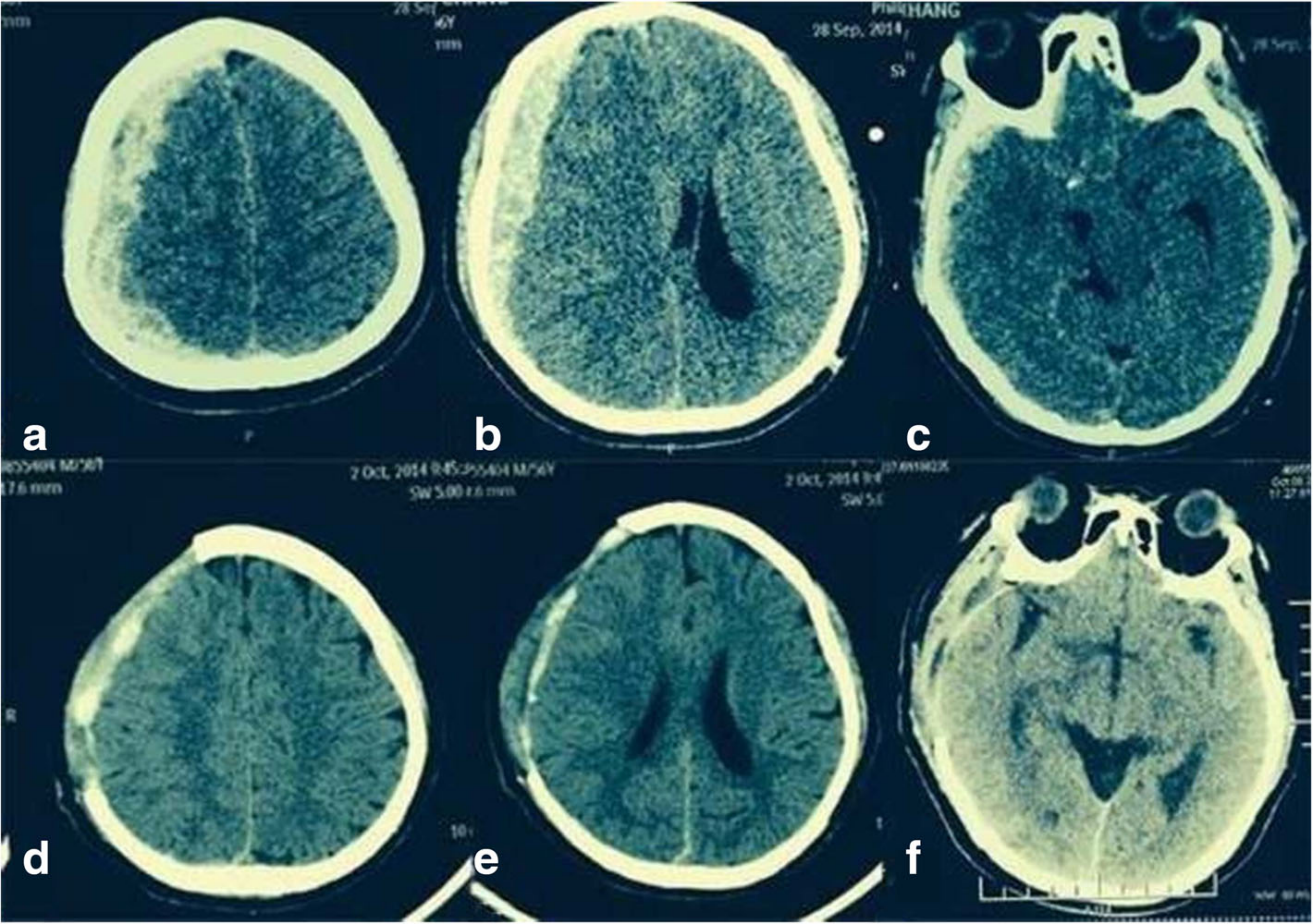
**a**, **b**, **c** CT scans before the operation. SDH was detected at the parietal and frontotemporal level of the right side. **d**, **e**, **f** CT scans after the operation. SDH had disappeared

The patient received a 250-ml infusion of mannitol as soon as he was admitted. We held consultations in the Hematology Department, intensive care unit, and Anesthesiology Department. We agreed that infusing sufficient factor VIII was the most important factor in the perioperative period. The operation proceeded normally, and the amount of bleeding was 4 U. Preoperative and postoperative CT scans are shown in Fig. 1. Based on the consultations, we infused the patient with 2400 U (40 U/kg) of factor VIII during the operation. We continued to infuse 2400 U of factor VIII per 12 h for the first 3 days after surgery, 1600 U per 12 h from the 4th day to the 1st week, and 1000 U per day from the 1st week to the 2nd week. The APTT was 34–57 s 2 weeks after the operation. When he was released from the hospital, factor VIII inhibitor was 0, and factor VIII activity was 25.9%. The APTT was 55.5 s, and the international normalized ratio was 1.16. The right oculomotor nerve showed paralysis with ptosis, and the diameter of the pupil was 5 mm. The right eye could abduct only. Myodynamia had recovered, and the Babinski sign was negative on both sides. He had a follow-up visit 3 months after discharge, and right oculomotor and right eye movements were observed. Cranioplasty was required after 6–12 months.

## Discussion

Hemophilia is an inherited bleeding disorder that can be classified into three types based on deficiency of the following coagulation factors: factor VIII (hemophilia A), factor IX (hemophilia B), and factor XI (hemophilia C). Hemophilia is classified as severe (factor level < 0.01 IU/ml), moderate (factor level < 0.05 IU/ml), and mild (factor level ≥ 0.05 and < 0.4). Hemophilia A, which occurs in approximately 1 in 5000 live male births, is more common and more likely to be severe, and morbidity is higher among males than among females. A prolonged APTT is a factor used to identify hemophilia. Primary treatment for hemophilia A is infusion of sufficient factor VIII and an emergency operation. Monitoring coagulation function is also important during the perioperative period.

Our patient was a middle-aged male who had inherited hemophilia A. He was admitted to the emergency room following a diagnosis of SDH. Because of his clinical history of hemophilia A and the severity of the disease, we measured his factor VIII activity levels in an APTT-based assay (APTT prolongation time). Normally, a prolonged APTT increases as hemophilia severity worsens. However, APTT might be normal in mild factor deficiencies. Patients with severe hemophilia rarely exhibit mild clinical signs [2] but can present with normal von Willebrand factor antigen levels, platelet counts, and prothrombin times.

Once the patient was admitted, normal treatment to reduce intracranial pressure was administered. A CT scan was performed upon his arrival to the hospital to evaluate his illness [3]. The diagnoses of SDH and hemophilia A were confirmed. Mild hemophilia is observed with bleeding in response to injury or surgery, such as bleeding of the teeth or mucous bleeding, mainly later in life. ICH and joint destruction are the two most important late complications observed patients with hemophilia. ICH, one of the most serious and life-threatening events, is relatively rare in patients with hemophilia. ICH can occur spontaneously or after trauma; it may also be caused by other risk factors (e.g., severe diseases and infection). Frequently, the pathogenesis of ICH is unknown [4, 5].

Because of its etiology, hemophilia A requires treatment with factor VIII. Prophylaxis is effective in decreasing the incidence risk of ICH [6, 7]. Since the 1960s, the mortality of ICH has declined from 70 to 20% [4]. When our patient was admitted, we administered an infusion of 2400 U (40 U/kg) of factor VIII. Rivera-Nunez et al. [3] suggested that a clotting factor should be provided as soon as possible and that the patient should be observed in the emergency department for at least 48 h after the operation. We immediately prepared for an operation. Fortunately, our hospital had sufficient factor VIII for infusion prior to the patient’s operation. Sufficient factor VIII and a short preoperative period decreased postoperative complications and improved the patient’s prognosis. The immediate treatment and operation were important for saving his life. Our patient’s factor level was not measured because of limited time, but his family mentioned that it was 0.029 IU/ml at the beginning of 2014. The diagnosis of hemophilia in patients with ICH is sometimes neglected, especially in newborns. Treatment and prognosis vary between hemophilia A and factor XIII deficiency. Therefore, a differential diagnosis between hemophilia and factor XIII deficiency is needed. Hemophilia and factor XIII deficiency can both present with bleeding in patients with ICH. Factor XIII deficiency is always detected by a normal APTT and prothrombin time, and factors VIII, IX, and XI exhibit normal levels. Factor VIII is located on the X chromosome, and factor VIII deficiency can be inherited or acquired. Therefore, genotyping may be another way to distinguish hemophilia and factor XIII deficiency.

Perioperative management of hemophilia is one of the most important steps to save patients’ lives, especially patients with a high-responding inhibitor, which is an antibody (primarily immunoglobulin G (IgG)). Approximately 25–30% of patients with severe hemophilia A have factor VIII inhibitors. We did not detect a factor VII inhibitor in our patient, and the effect of factor VIII was obvious. If a hemophilia patient has an inhibitor, we can use an Aminco Continuous Flow Celltrifuge; perform immunosuppression with.

Cyclophosphamide; and administer corticosteroids, ε-aminocaproic acid, and large doses of antihemophilic globulin (AHG) concentrates [8]. However, these treatments may lead to complications such as hemolysis and platelet dysfunction because of hyperfibrinogenemia, leukopenia, and Gram-negative sepsis. We infused our patient with 200 ml fresh frozen plasma on the 1st postoperative day to increase blood capacity. However, plasma infusion alone may not decrease the danger of bleeding. Infusing only fresh frozen plasma may increase the risk of hemorrhagic infarction and acute SDH, which will require additional operation(s) [9]. We monitored the level of factor VIII activity 10 days after the operation, and it was 25.9%. A total of 30% of plasma VIII factor is sufficient for preventing ICH in patients with hemophilia [10]. However, bleeding with a lower factor VIII activity level could still be controlled in our patient. Our patient was discharged after receiving infusions of factor VIII for 2 weeks, and the APTT was 34–57 s. Oculomotor paralysis remained when he was discharged from the hospital, and a follow-up clinic was needed. Titanium mesh reconstruction of a right bone flap was considered necessary after 3–6 months. Hemophilia in addition to a nervous system syndrome should be controlled by a follow-up clinic. Nutrient support was one of the most important factors in the postoperative recovery of our patient. Our patient was supported by a semi-liquid diet via a nasogastric tube during the first 2 postoperative days before he could eat by himself.

## Conclusion

We treated our patient with a high-quality infusion of factor VIII, which helped save valuable time and improve his prognosis. Therefore, factor VIII infusion is the most important factor in treating SDH patients with a medical history of hemophilia A. We recommend monitoring coagulation function and determining the medical history once the patient is admitted, especially in the emergency department.

## Abbreviations

AHG: Antihemophilic globulin
APTT: Activated partial thromboplastin time
CT: Computed tomography
ICH: Intracranial hemorrhage
SAH: Subarachnoid hemorrhage
SDH: Subdural hematoma
